# Guiding point-of-care therapeutic drug monitoring through structure–toxicity principles

**DOI:** 10.1039/d6sc03923e

**Published:** 2026-07-02

**Authors:** Jiarui Wang, Lingling Guo, Chuanlai Xu, Aihua Qu, Hua Kuang, Xinxin Xu

**Affiliations:** a International Joint Research Laboratory for Biointerface and Biodetection, Jiangnan University Wuxi Jiangsu 214122 China xuxinxin@jiangnan.edu.cn kuangh@jiangnan.edu.cn

## Abstract

A structure–toxicity strategy was employed to generate monoclonal antibodies exhibiting a gradient of affinity corresponding to the toxicity level of biguanides, and an immunochromatographic sensor was established for diabetes drug risk monitoring, enabling a therapeutic drug monitoring function that can accurately identify high-risk biguanides in human serum. The data demonstrated that the H1-induced monoclonal antibody 2G8 was generated with the ability to recognize biguanides according to the toxicity gradient (affinity gradient: phenformin > buformin > moroxydine > metformin), with IC_50_ values ranging from 0.11 to 3.35 ng mL^−1^. Molecular docking revealed that the high affinity was primarily driven by π–π stacking interactions between H: PHE 55/L: PRO 116 residues and biguanides. The developed immunochromatographic sensor for biguanides achieves a cut-off value of 2–500 ng mL^−1^ in human serum. Furthermore, based on affinity gradient properties, this sensor can both accurately monitor adherence to metformin therapy in diabetic serum (500–5000 ng mL^−1^) and provide rapid visual early warning of metformin overdose (20 µg mL^−1^) and trace phenformin toxicity (200 ng mL^−1^). Remarkably, the generalizability of the structure–toxicity principle was well validated in structurally complex macrolides. The developed method opened an avenue for coming up with a comprehensive risk management strategy encompassing clinical monitoring and poisoning risk assessment.

## Abstract 2

A structure–toxicity strategy was employed to generate monoclonal antibodies exhibiting a gradient of affinity corresponding to the toxicity level of biguanides, and an immunochromatographic sensor was established for diabetes drug risk monitoring.

## Graphical abstract

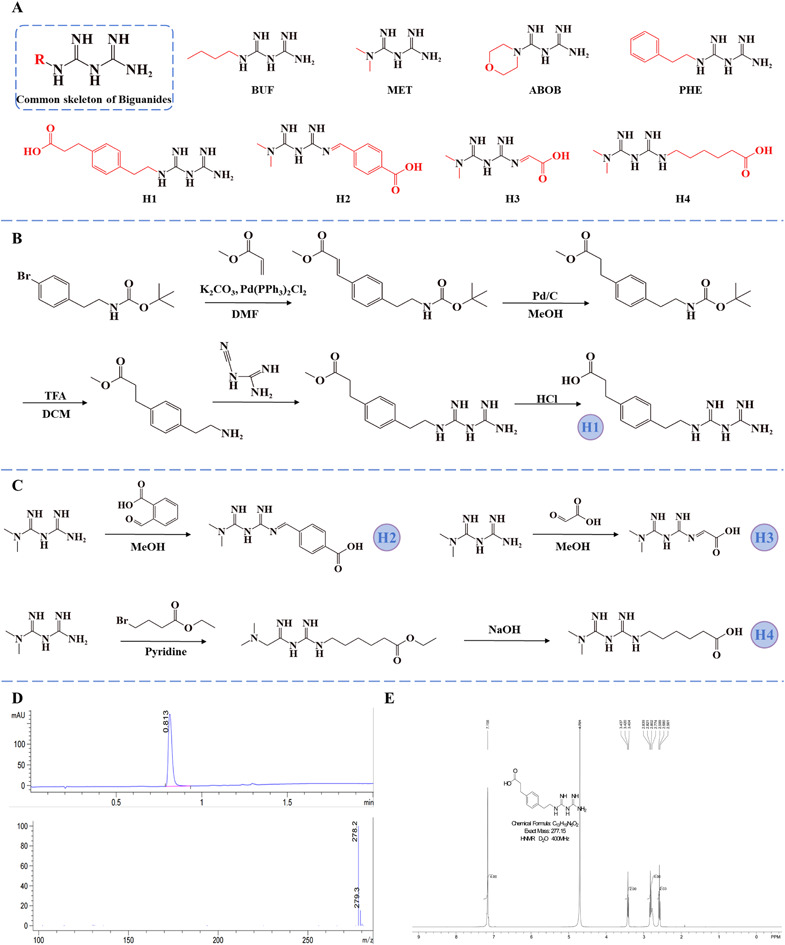

## Introduction

Biguanides, including phenformin (PHE), buformin (BUF), metformin (MET), and moroxydine (ABOB), share a common guanidino core structure.^1,2^ MET, PHE, and BUF have been widely used as oral hypoglycemic agents.^3^ Among them, MET has become the preferred drug for treating type 2 diabetes mellitus worldwide due to its low toxicity.^4–6^ Although certain biguanides (such as BUF and PHE) are no longer routinely used in many countries due to safety concerns, they remain of significant interest in toxicological and medical research and continue to be important targets for detection in the analysis of biological samples.^7–9^ Therefore, risk screening for biguanides in clinical diagnostics remains a serious challenge. The risk of exposure to highly toxic biguanides remains, mainly from unregulated routes of intake, such as health supplements that claim to have fast-acting glucose-lowering properties.^10^ There is also the possibility that ABOB with antiviral activity could be illegally used as health supplements, thus threatening human health.^11,12^ It is urgently important to develop a method that can simultaneously and rapidly screen these four biguanides to achieve timely warning of the risk of toxicity in patients. Studies have shown that the mitochondrial toxicity of biguanides is closely related to the physicochemical properties of their guanidino side chains; the more complex and lipophilic the side chain structure, the higher the potential toxicity tends to be.^13^ This indicates that the ideal clinical diagnostic tool is not only a broad-spectrum identification of drugs; it should also possess a differential sensitivity that matches the toxicity level of the drugs.

In therapeutic drug monitoring (TDM), maintaining therapeutic drug concentrations within the therapeutic window is crucial.^14–17^ This involves monitoring patient adherence to prevent underexposure and providing rapid early warning for drug overdose or toxicity.^18–20^ Although instrumental analysis and electrochemical methods allow for precise quantification, they typically require specialized equipment and are therefore not well-suited for rapid risk assessment in routine clinical practice.^21–24^ Immunochromatographic assays (ICAs) provide an ideal solution for this purpose.^25–28^ However, conventional ICA does not allow for gradient-type identification based on the toxicity level of the analyte.^29–31^ The existing broad-spectrum monoclonal antibody (mAb) typically shows equal affinity for all analytes,^14,32–34^ which makes it difficult to achieve accurate risk monitoring in complex matrices containing high concentrations of low-toxicity drugs, and it is difficult to accurately identify illegally added trace amounts of highly toxic drugs, which can easily lead to misclassification of results.

The preparation of monoclonal antibodies capable of broad-spectrum recognition based on analyte toxicity gradients is challenging. To address this challenge, we used a structure–toxicity oriented computer-assisted hapten design strategy.^35,36^ Differences in toxicity based on biguanides are closely related to their diverse side-chain structures (highly toxic PHE contains hydrophobic phenylethyl groups, whereas low-toxicity MET contains hydrophilic short-chain alkyl groups); PHE and MET were chosen as design templates with the aim of screening mAb with affinity gradients. Finally, we analyzed the recognition mechanism of mAb by molecular docking techniques.^37–39^ The results showed that the screened mAb-2G8 exhibited the expected affinity gradient: PHE > BUF > ABOB > MET. And we further developed the ICA method, and validate the effectiveness and feasibility of our customized hapten design strategy at the application level. Moreover, monitoring the risk of biguanide adherence and poisoning in the context of diabetic serum was successfully achieved, which exploits the difference in affinity of antibodies for the analyte, validating the feasibility of this strategy in practical applications. Finally, we investigated whether the affinity gradient pattern observed in simple linear biguanide molecules could be extended to more structurally complex macrocyclic lactone compounds, thereby evaluating the broader applicability of this design strategy.

## Results and discussion

### Computer simulation for design of biguanide haptens

**Fig. 1:**
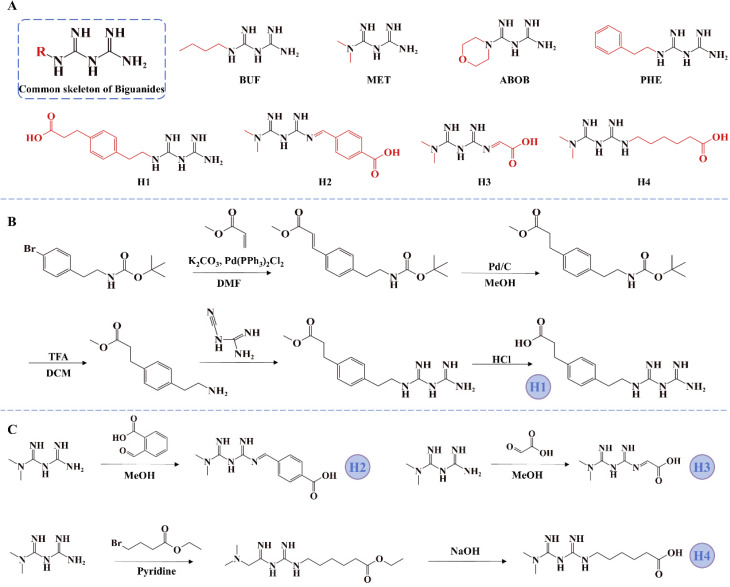
Design, synthesis and structural characterization of biguanide haptens. (A) 2D structure of 4 biguanides and haptens, (B) synthesis pathways of H1, and (C) synthesis pathways of H2, H3, and H4.

In the immunological detection of biguanide drugs, the precise construction of hapten structures is a critical step for generating antibodies with high specificity and affinity.^40–42^ The design of haptens was guided by the structure–toxicity strategy in order to obtain a broad-spectrum recognising antibody that matches the toxicity. It has been shown that the toxicity of biguanides tends to be positively correlated with their lipophilicity.^43^ Therefore, the focus of this process is to maintain the major structural characteristics of the parent compound but to vary the molecular lipophilicity to ensure that immunological recognition aligns with the principle of toxicity matching. The active site for binding to carrier proteins is generated by the addition of carboxyl group-containing spacer arms to efficiently stimulate an immune response. In the hapten design, we chose PHE and MET as templates, representing relatively hydrophilic and more lipophilic structural features, respectively. And the differential side-chain structures of the two set the stage for building antibody sensitivity that matches toxicity. The four haptens were designed as follows: a flexible spacer arm containing a terminal carboxyl group was introduced at the *para*-position of the benzene ring of PHE, and a carboxyl-containing spacer arm (containing an aromatic ring, a short carbon chain, and a long carbon chain) was introduced at the guanidinium terminus of MET (Fig. 1).

**Fig. 2:**
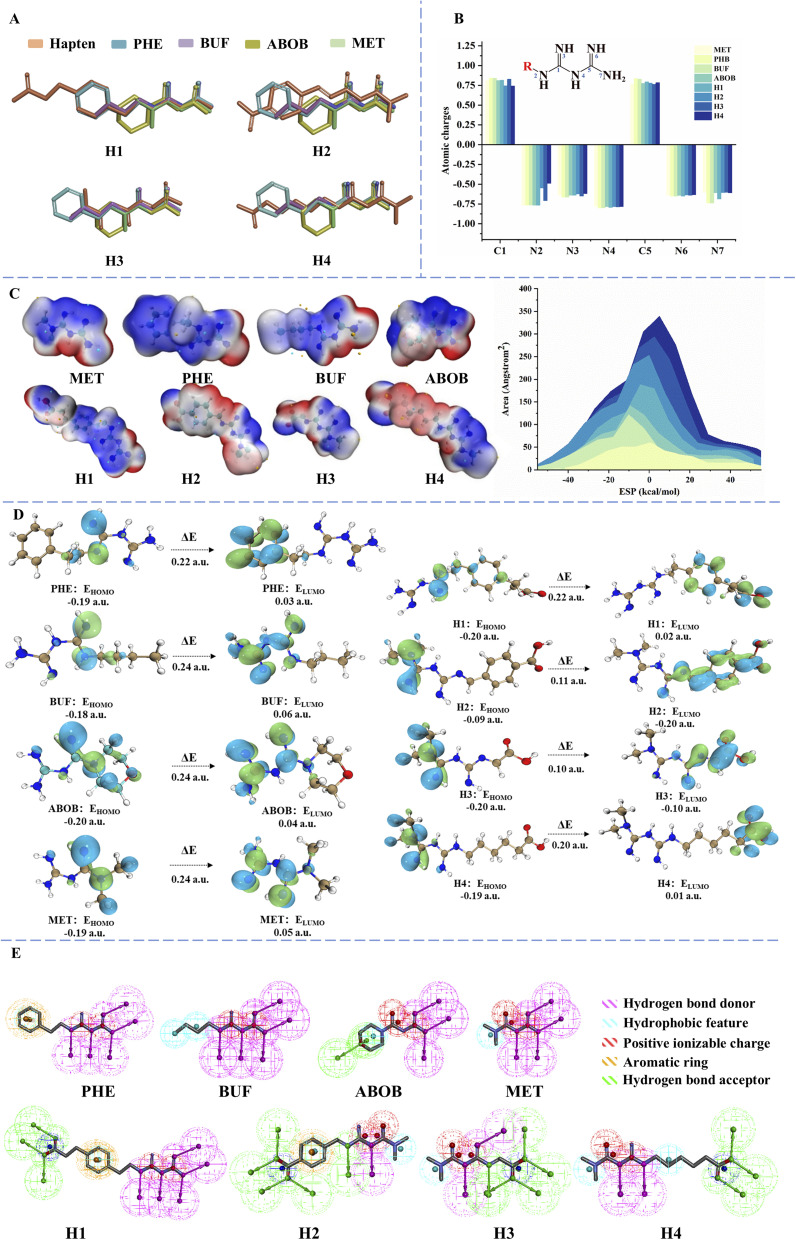
Hapten design and computer-aided analysis results. (A) The steric overlap of biguanides and haptens, (B) charge distribution analysis, (C) ESP surface distribution and surface area in each ESP range, (D) plots of frontier molecular orbitals, *E*_HOMO_ and *E*_LUMO_, and (E) the pharmacophore analysis of biguanides and haptens.

Four haptens were evaluated using computerised chemical prediction using five descriptors: molecular overlap, atomic charge distribution, electrostatic potential (ESP), molecular orbitals and pharmacophore characteristics. The three-dimensional conformations of biguanides and haptens showed different degrees of overlap (Fig. 2A). Notably, H1 showed the highest degree of overlap similarity with the biguanides, followed by H3 and H4 (the latter two do not contain aromatic rings). This difference suggests that the introduction of a spacer arm into the prodrug structure would significantly induce conformational deflection of its epitope, thereby reducing the conformational similarity between the molecules. As shown in Fig. 2B, the visualisation of atomic charge distribution revealed that both the target biguanide molecules and their corresponding haptens, H1–H4, exhibited highly consistent charge characteristics in the biguanide parent core region. This is a direct indication that the hapten design successfully mimics the electron distribution of the target on this key pharmacophore. ESP analysis showed that H1, H3 and H4 retained the core characteristics of biguanides, with ESP values ranging from −40 to 40 kcal mol^−1^ (Fig. 2C). H2 exhibited stronger positive ESP, but biguanides showed stable negative ESP around the guanidinium group.

In addition, electronic properties are another key determinant of the immunogenicity of the haptens. Molecular orbital analysis shows that the lowest unoccupied molecular orbitals (LUMOs) of the non-aromatic drugs BUF, MET and ABOB are mainly localised in the protonated biguanide backbone, a feature that facilitates the formation of strong ionic bonds with negatively charged amino acids in the complementarity-determining region (CDR) of the antibody and the construction of an interaction network with multiple hydrogen bond donors/acceptors, resulting in high affinity (Fig. 2D). In contrast, the LUMO of PHE is distributed on both the biguanide core structure and its aromatic ring, which enables PHE to generate significant π–π stacking and hydrophobic interactions with aromatic amino acids in the CDR region of the antibody, inducing higher affinity. On the other hand, the HOMO–LUMO energy gap (Δ*E*) reflects the kinetic stability of the molecule, and higher Δ*E* values usually imply greater stability.^44,45^ The results show that the Δ*E* values of H1 and H4 are the closest to those of the analytes, indicating better stability.

To thoroughly analyze the structure–function relationship and predict the binding mode between haptens and analytes, we conducted a pharmacophore analysis (Fig. 2E). First, for the analytes, their shared biguanide core structure showed the same pharmacophore pattern, containing two positive ionization centers (red spheres) and nine potential hydrogen bond donors (pink spheres). Significantly, this feature was perfectly preserved in all haptens, suggesting that the addition of spacer arms to either the benzene ring counterpart (H1) or the biguanide tail (H2–H4) did not affect recognition of the analyte’s broad-spectrum backbone. Secondly, the variability in the side-chain structure of the analytes gives some differences in their pharmacophore profiles based on their common possession of hydrophobic characteristic groups. PHE possesses an aromatic ring, whereas ABOB possesses a morpholine ring containing a potential hydrogen bond acceptor.

The pharmacophore profile of H1 was the best match to the analyte. H2, although possessing fewer hydrogen bond donors, shares an aromatic ring with H1, which may allow them to be recognized *via* π–π stacking interactions during immunization, thus inducing the production of high affinity antibodies. In contrast, H3 and H4, which contain aliphatic carboxyl chains, lack aromaticity and adopt conformations similar to the low-toxicity MET. These results suggest that the haptens designed in this study can effectively match the structural diversity of the analytes, allowing the final antibodies to both recognize the common backbone and produce differences in sensitivity.

### Mouse antiserum and cell line screening

**Fig. 3:**
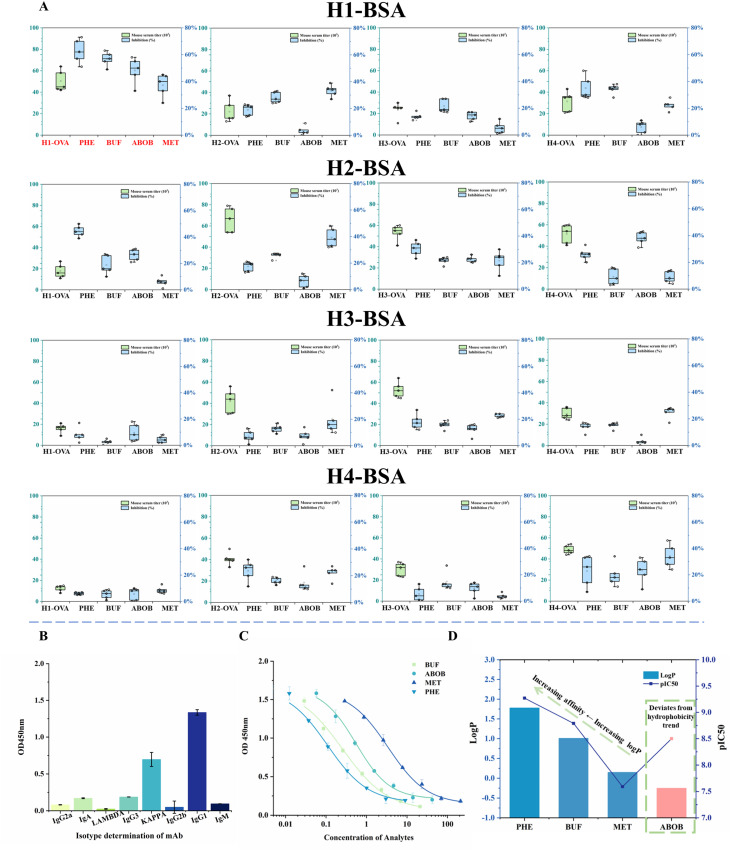
Preparation, characterization and performance analysis of mAb. (A) Screening of immunogens and coating agents: mouse serum antibody titer (green) and the inhibition rate against target analytes (blue), (B) isotype identification, (C) ic-ELISA standard curve, and (D) the correlation between antibody affinity (pIC_50_) and analyte lipophilicity (log *P*).

We synthesized H1–H4 and prepared artificial antigens for mouse immunization experiments; the results of characterization of haptens and artificial antigens are shown in Fig. S1–S3 and Table S1. The high inhibition rate indicates that the mouse serum antibodies exhibit a strong specific recognition of the target chemical residues, reflecting their high sensitivity. Elevated serum titres indicate high affinity of the antibody. In this study, four immunogens (H1-BSA–H4-BSA) were synthesized and used in mouse immunization experiments, with their immunogenicity assessed after booster immunization. We concurrently synthesised the corresponding four coating antigens. Through paired screening, we found that the homologous coating model (where both the immunogen and coating antigen were derived from the same hapten) exhibited optimal detection performance across all combinations. As shown in Fig. 3A, homologous coating induced high serum antibody titres in all four mouse groups. Although mice immunised with H2-BSA exhibited the highest antiserum titre under the homologous coating, it only achieved an inhibition rate exceeding 40% for MET, failing to meet the requirements for broad-spectrum recognition. Notably, serum from H1-BSA-immunised mice exhibited high rates of inhibition of all four biguanides and showed the gradient structure that we expected to match toxicity. Ultimately, immunised mice with serum titres of 6.4 × 10^4^ and inhibition rates of 73% (PHE = 5 ng mL^−1^), 57% (BUF = 10 ng mL^−1^), 58% (ABOB = 20 ng mL^−1^), and 40% (MET = 100 ng mL^−1^) were selected for splenocyte fusion, serving as the basis for subsequent mAb preparation.

### Characterization of the mAbs

The mAb-2G8 obtained in this study has been subclass identified as IgG1, Kappa (Fig. 3B). Fig. S4 shows that the antibody affinity constant (*K*_a_) value of mAb-2G8 prepared in this study was 2.67 × 10^9^ L mol^−1^. A standard curve was established by ic-ELISA to investigate the sensitivity of mAb-2G8. As shown in Fig. 3C, the IC_50_ values of this antibody for PHE, BUF, ABOB and MET were 0.11 ng mL^−1^, 0.25 ng mL^−1^, 0.54 ng mL^−1^ and 3.35 ng mL^−1^, respectively.

In this study, log *P* was used as a parameter reflecting the lipid solubility of analytes to indirectly assess the relative toxicity risk of different analytes. To examine the relationship between the sensitivity of mAb-2G8 and the toxicity of the analytes, the −log IC_50_ value (pIC_50_) of each analyte was plotted against its toxicity index (log *P*). Fig. 3D shows that PHE, BUF and MET together form a decreasing trend from high log *P*, high affinity to low log *P*, low affinity, demonstrating that the mAb obtained in this study has an affinity gradient that matches the toxicity of the analyte. And the hydrophobic effect is likely to be the dominant force that enhances the recognition and binding stability of the antibody. In addition, ABOB significantly deviates from this trend, and although it does not contain a hydrophobic moiety, the morpholine ring contains potential hydrogen-bonded receptor sites that may also drive highly sensitive recognition of it by antibodies. Polar heterocycles and the stereo conformation may generate additional interactions (*e.g.*, hydrogen bonds), thereby enhancing binding affinity. It should be noted that log *P* solely reflects a compound's hydrophobic properties, exhibiting a correlation with toxicity but not a determinative relationship. Toxicity levels remain influenced by other structural factors, including polarity distribution.

For four biguanide compounds (Table S2), the cross-reactivity of mAb-2G8 was evaluated. As anticipated based on our toxicity-matched hapten design, the antibody exhibited a distinct affinity gradient: the most toxic analogue PHE demonstrated the strongest binding affinity, whilst the least toxic analogue MET exhibited the lowest affinity.

### Molecular modeling and the recognition mechanism

Since the affinity gradient of the experiment (PHE > BUF > ABOB > MET) was observed, we needed a highly qualitative 3D structure of the mAb to analyze the direct interactions between the ligand and the receptor. Ramachandran plots and Verify3D profiles were used to determine the structural integrity of the homology model.^46,47^ As shown by the Ramachandran plot (Fig. S5), the percentage of residues in favorable or allowed regions (99.0% of the total) was very large, indicating a reasonable protein structure. The agreement between the amino acid sequence and tertiary structure was also confirmed by Verify3D (Fig. S6) with 86.58% of the residues having a score of 0.1 or above. Such high structural reliability provides a robust foundation for subsequent molecular docking analysis.

**Fig. 4:**
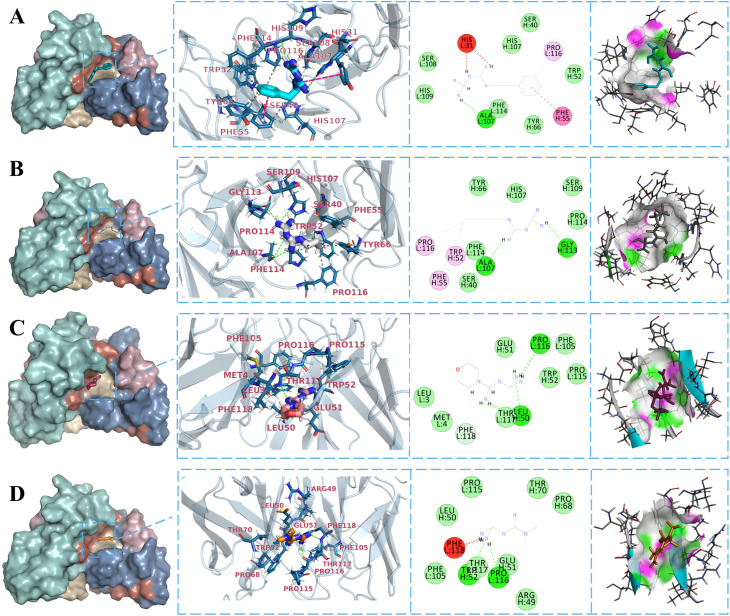
Molecular docking analysis of the interactions between mAb-2G8 and four biguanides. The rows represent the docking results for (A) PHE, (B) BUF, (C) ABOB, and (D) MET, arranged in descending order of binding affinity. (Left panel) Surface representation showing the ligand embedding into the binding pocket. (Middle-left panel) 3D visualization of the binding site, identifying key interacting residues. (Middle-right panel) 2D interaction maps. Green dashed lines indicate hydrogen bonds; pink/purple lines indicate hydrophobic interactions (π–π and π-alkyl); red lines indicate unfavorable hydrogen bond donor–donor repulsion. (Right panel) Hydrogen bond surface mapping of the binding pocket accommodating the ligands.

Molecular docking simulations were performed to determine the structural basis of the broad-spectrum recognition and toxicity matched sensitivity of mAb-2G8.^48^ As shown in Fig. 4, all four analytes (PHE, BUF, ABOB, and MET) docked into a common cleft formed by the CDRs, validating the hapten design strategy that preserved the biguanide scaffold.^49,50^

Calculated binding free energies (Δ*G*) followed the order PHE (−7.2 kcal mol^−1^) < BUF (−5.9 kcal mol^−1^) < ABOB (−5.5 kcal mol^−1^) < MET (−5.2 kcal mol^−1^). The gradient correlates well with experimental IC_50_ values (0.11–3.35 ng mL^−1^). PHE formed the most stable complex, consistent with its high toxicity, while the higher Δ*G* of MET accounts for its lower, yet sufficient, binding affinity (IC_50_ = 3.35 ng mL^−1^).

A conserved interaction network anchors the shared biguanide core, primarily involving residues H: TRP 52, L: PRO 116, H: PHE 55, and L: ALA 107. The analytes produce π–π stacking and π-alkyl interactions with H: PHE 55 and L: PRO 116, while L: ALA 107 and H: TRP 52 hydrogen bond with the guanidinium backbone of the analytes.

Through molecular interaction analysis, we explored the driving forces of the affinity gradient gap. The results show that the affinity level is dominated by hydrophobic interactions of the analyte with residues in the binding pocket, while van der Waals and hydrogen bonding interactions ensure a broad-spectrum recognition pattern of the antibody.^51^ This is consistent with the toxicity gradient of the analytes. PHE (log *P* = 1.78) with the highest lipophilicity and toxicity exhibited the highest binding affinity in the binding pocket (Fig. 4A). The phenylethyl group of PHE formed strong π–π stacking interactions with H: PHE 55 and π-alkyl interactions with L: PRO 116, resulting in the highest affinity. Interestingly, the 2D and 3D results differ on PHE interaction with L: HIS 31 (2D: unfavorable hydrogen bond donor–donor repulsion; 3D: hydrogen bonding). This difference suggests that the tautomerism and conformational flexibility of L: HIS 31 may alleviate steric hindrance in solution, leading to stable interactions. Overall, these interactions give mAb-2G8 the highest affinity and detection sensitivity for PHE.

BUF exhibits lower toxicity. Fig. 4B shows that although its butyl side chain partially forms π-alkyl interactions with L: PRO 116, H: TRP 52 and H: PHE 55, the lack of an aromatic ring that undergoes π–π stacking interactions with H: PHE 55 results in a hydrophobic network that is not as intact as that of PHE with a weakened affinity. ABOB, on the other hand, showed a different binding form (Fig. 4C). Unlike the hydrophobic interaction of PHE and BUF, it binds to antibodies mainly through hydrogen bonding with H: LEU 50 and L: PRO 116. Due to steric hindrance, the strongly polar morpholine ring of ABOB could not penetrate deeper into the binding pocket, which resulted in a weaker binding capacity. The low log *P* value of ABOB became an outlier in the affinity-toxicity gradient (Fig. 4D), which suggests that mAb-2G8 does not rely on hydrophobic interactions alone to generate a strong recognition mechanism, and that polar interactions such as hydrogen bonding may also induce a strong binding capacity.

MET has the simplest structure and low lipophilicity (log *P* = 0.15). As shown in Fig. 4D, the short methyl side chain is unable to form hydrophobic interactions and relies primarily on hydrogen bonding interactions with H: TRP 52 and L: PRO 116. In addition, the 2D plot shows that there is an unfavorable hydrogen bond donor–donor repulsion between MET and L: PHE 118, which further reduces the stability of the binding, a result that is consistent with its lowest toxicity and weakest affinity.

### Optimization and application of the ICA sensors

**Fig. 5:**
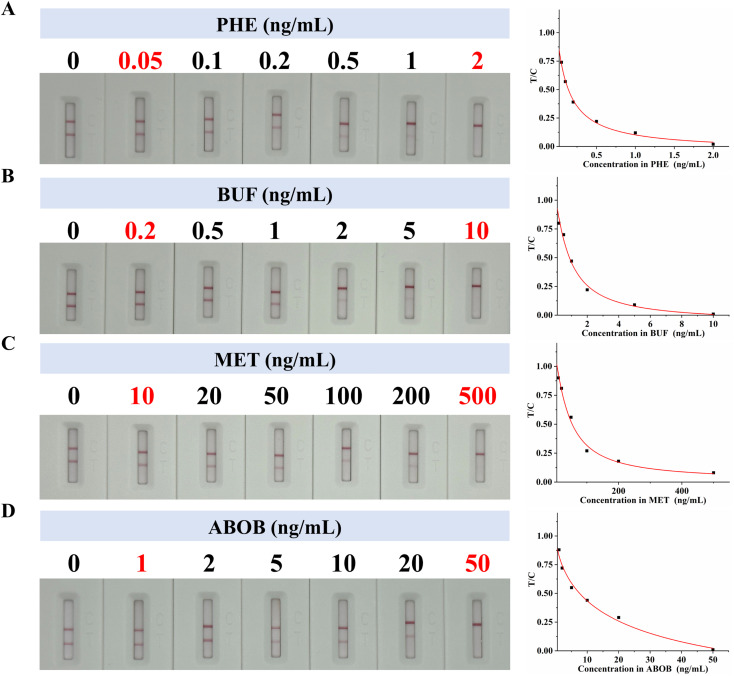
The ICA sensor for the determination of spiked biguanides in negative human serum samples. (A) PHE, (B) BUF, (C) MET, and (D) ABOB.

The performance of the ICA sensor is affected by the amount of GNPs-mAb and the concentration of the coating antigen, which may affect the accuracy of the experiment if the concentration is too high or too low.^52^ In this study, the amounts of GNPs-mAb and coated antigen at different concentrations were optimized to obtain the best experimental conditions. The results are shown in Fig. S7. The optimal amount of GNP-mAbs was 5 µg mL^−1^, and the optimal concentration of the coating antigen was 0.02 mg mL^−1^. Selection of a suitable gold standard resuspension is critical for stabilizing antibody activity and reducing non-specific adsorption.^53^ In this study, four common resuspensions were selected for optimization: 5% PVP, 5% ON-870, 5% R65, and 5% BSA (Fig. S7), and the results indicate that a 5% PVP solution is optimal in terms of color intensity and inhibition rate (84.8%). Following optimization of the conditions, various concentrations of PHE, BUF, ABOB, and MET were added to PBS (0.01 mol L^−1^, pH 7.4) to examine the sensitivity of the sensor (Fig. S8). At the same time, to evaluate the sensitivity of ICA in human serum, we performed ICA assays using blank human serum samples spiked with PHE, BUF, ABOB, and MET, as shown in Fig. 5. And the standard curve equations of ICA in human serum samples are shown in Table S4. We define the visual limit of detection (vLOD) as the minimum target concentration at which the *T*-line color is clearly lighter than the negative control (*i.e.*, exhibiting a pale red hue) upon visual inspection. The minimum target concentration required to completely eliminate the *T*-line color is termed the cut-off value. The vLOD values in human serum were 0.05 ng mL^−1^, 0.2 ng mL^−1^, 1 ng mL^−1^, and 10 ng mL^−1^ respectively. And the cut-off values were 2 ng mL^−1^, 10 ng mL^−1^, 50 ng mL^−1^, and 500 ng mL^−1^, respectively. The ICA cut-off values in PBS were 1 ng mL^−1^, 5 ng mL^−1^, 10 ng mL^−1^, and 100 ng mL^−1^ respectively. To assess the risk of false positives in a true negative matrix, an additional 40 samples of normal human serum were tested. The results showed that all 40 unspiked normal human serum samples were classified as negative (Fig. S9), with no false-positive results observed. This indicates that the method exhibits good specificity and a low risk of false positives in normal human serum samples.

### ICA sensor for clinical risk screening methods in diabetic patients

Human serum samples were obtained from the Affiliated Hospital of Jiangnan University. This study was conducted in accordance with the Declaration of Helsinki and approved by the Ethics Committee of Affiliated Hospital of Jiangnan University (Approval No. LS2024356). All experiments were conducted in accordance with the guidelines of the Ethics Committee of the Affiliated Hospital of Jiangnan University. Informed consent was obtained from all individual participants included in the study.

**Fig. 6:**
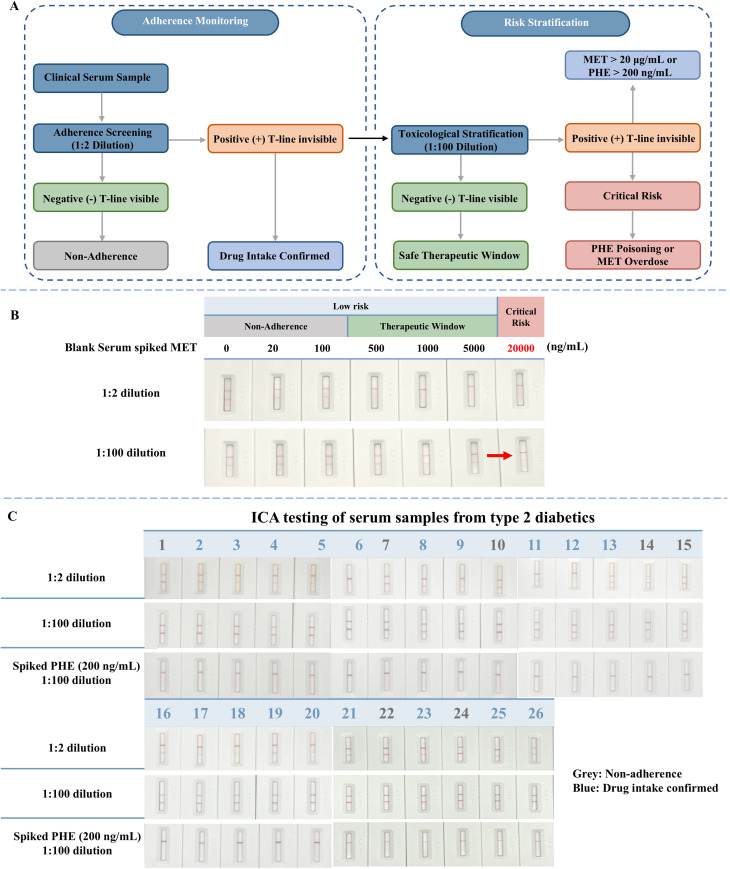
Establishment and application of a clinical risk monitoring process for biguanides in human serum. (A) The clinical risk monitoring process for MET and PHE in human serum, (B) the results of testing of blank serum spiked with different concentrations of MET, and (C) serum ICA sensor results from real type 2 diabetes patients (sample 1 to sample 26, with 1 : 2 dilution and 1 : 100 dilution), and simulated PHE poisoning ICA test results (1 : 100 dilution).

To realize the application of ICA for therapeutic drug monitoring and toxicity risk screening in type 2 diabetic patients within a clinical setting, this study established a simple dilution detection strategy utilizing the unique affinity gradient feature of the mAb-2G8, and the operation flow is summarized in Fig. 6A. In standard clinical practice, MET concentrations in human blood should be maintained between 0.5 and 3 mg L^−1^.^54^ We evaluated the sensitivity of the ICA using a blank serum spiking experiment (Fig. 6B). At a 1 : 2 dilution ratio, the test line (*T*-line) signal completely disappeared when MET concentrations exceeded 500 ng mL^−1^. This indicates that, at this dilution ratio, the test can reliably determine whether a patient is adhering to the prescribed medication regimen. However, this approach fails to differentiate between normal treatment levels and drug accumulation. Therefore, we introduced a high dilution factor (1 : 100) to visually determine whether a serum concentration falls outside the safety window by observing the disappearance of the *T*-line in the ICA. At a 1 : 100 dilution, MET signals became negative at typical concentrations, and the *T*-line disappeared only when the MET concentration reached a high-risk accumulation level (20 000 ng mL^−1^). This indicates that the 1 : 100 dilution effectively establishes a safety window for quantitative analysis.

Serum samples were collected from 26 patients with type 2 diabetes treated with MET and with no history of other drug exposure. These samples were tested after a 1 : 2 dilution (Fig. 6C). The results showed that 19 patient samples produced distinct positive signals, confirming the capability of ICA to effectively detect MET in patients. In TDM clinical practice, this step can rapidly identify patients who are non-compliant or metabolize the drug too quickly (7 patients presented with MET non-adherence), thereby assisting physicians in assessing medication adherence. Furthermore, to screen patients for potential hypertoxic PHE, we conducted a secondary screening of the aforementioned clinical samples at 1 : 100 dilution. The results showed that all patient samples tested negative at 1 : 100 dilution (*T*-line clearly visible), demonstrating that none of the patients were at serious risk of MET accumulation. Meanwhile, samples spiked with 200 ng per mL PHE still produced a positive ICA response after 100-fold dilution. This finding demonstrates that the sensor retains its ability to detect highly toxic prohibited substances even when high levels of MET are excluded, ensuring that no potential toxicity risk is overlooked. However, this strategy is designed for rapid risk screening rather than to definitively distinguish between MET overdose and illicit PHE exposure based solely on a single ICA test result, and suspicious samples identified through screening should be further confirmed by LC-MS/MS.

Combining the results of the above experiments, we established a clinical risk monitoring process for biguanides in human serum. This strategy enables comprehensive monitoring of medication safety in patients with type 2 diabetes through simple dilution modulation. At the same time, it should be noted that the use of multiple medications and the sample matrix in real-world clinical settings is highly complex, and the current sample size and scope of validation are insufficient to account for all possible coexisting interferences. Therefore, this method is currently best suited as a tool for rapid clinical screening and preliminary risk assessment.

### The universality of the structure–toxicity strategy from simple chains to complex rings

**Fig. 7:**
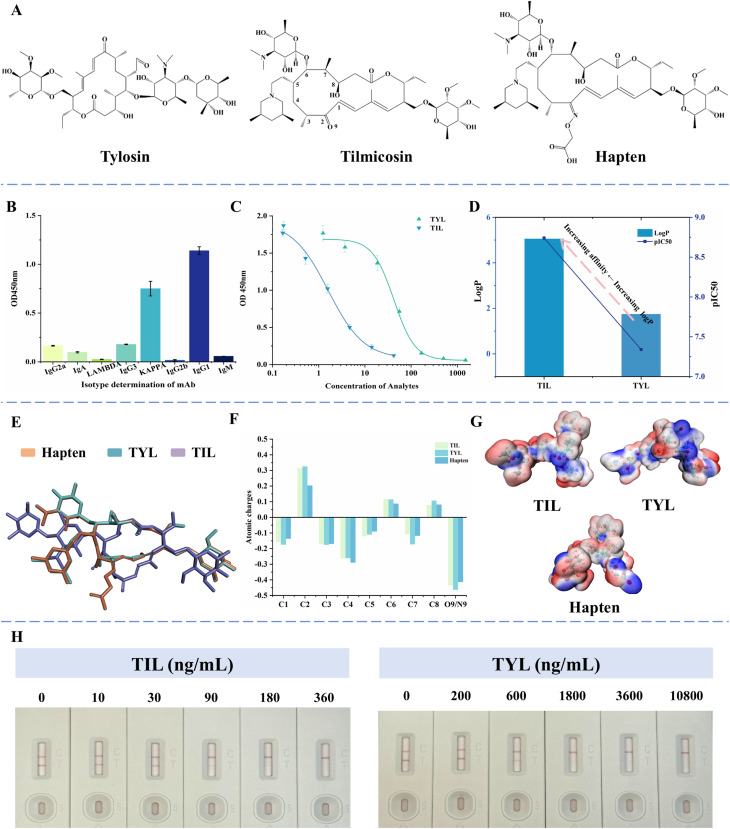
Development of TIL and TYL mAb based on the structure–toxicity strategy. (A) 2D structure of TIL, TYL and hapten, (B) isotype identification, (C) ic-ELISA standard curve, (D) the correlation between antibody affinity (pIC_50_) and analyte lipophilicity (log *P*), (E) the steric overlap of biguanides and haptens, (F) charge distribution analysis, (G) ESP surface distribution, and (H) the ICA sensor for the determination of spiked TIL and TIL in negative human serum samples.

To verify the generalizability of the structure–toxicity strategy for obtaining affinity-gradient antibodies targeting biguanides in this study, we extended our investigation from simple linear molecules to complex macrocyclic structures. As an example, we examined tilmicosin (TIL), which carries a high risk of cardiotoxicity, and its low toxicity analogue, tylosin (TYL). The primary difference between TIL and TYL is that TIL contains a unique lipophilic moiety, the 3,5-dimethylpiperidine ring. To maximize the exposure of this moiety to the hapten, the distal keto group was selected for oxime modification in this work (Fig. 7A). The results were subsequently validated through animal experiments and computational analysis. We synthesized a hapten and prepared an artificial antigen for mouse immunization experiments, ultimately obtaining the mAb-1B3. The characterization results of the hapten and the artificial antigen are shown in Figure S10. The isotype of mAb-1B3 is IgG1, kappa (Fig. 7B). The IC_50_ values of the antibody for TIL and TYL were 1.59 ng mL^−1^ and 42.22 ng mL^−1^, respectively, as shown in Fig. 7C. As illustrated in Fig. 7D, the affinity trends of TIL and TYL correlated synergistically with the lipophilicity of the molecules, supporting the validity of the structure–toxicity design strategy proposed in this study.

Through computer-assisted analyses, we elucidated the mechanism by which mAb-1B3 generates an affinity gradient (TIL > TYL). As shown in Fig. 7E, the 16 membered macrocyclic backbone and the peripheral glycan ring portion exhibit extremely high conformational similarity. Additionally, the hapten retains the key characteristic motifs of TIL intact, resulting in a high degree of conformational overlap in this region. The atomic charge distribution (Fig. 7F) shows that the electron cloud arrangement of the three molecules in the C1–C6 macrocyclic backbone is highly consistent. This enables the antibody to effectively recognize the shared backbone of TIL and TYL, thus explaining the antibody's recognition of TYL. Despite the backbone similarity, the hapten was designed to maximally preserve the ESP distribution of TIL through targeted modification at the C-2 position. As shown in Fig. 7G, the hapten and TIL exhibit a highly coincident ESP feature distribution in the lipophilic 3,5-dimethylpiperidine ring region. This differentiated ESP can explain the affinity gradient produced by the antibody for TIL and TYL.

To further assess the practical application potential of this design strategy, we developed an ICA sensor using mAb-1B3 for human serum assays (Fig. S11 for the optimization of the ICA sensor). As shown in Fig. 7H, the performance of the sensor in a serum matrix not only validates the reliability of the strategy but also demonstrates the broad applicability of the structure–toxicity principles presented in this work for complex biomonitoring.

## Conclusions

In this study, an ICA method based on computer-assisted hapten design was developed for the simultaneous detection of PHE, BUF, MET and ABOB. mAb-2G8 obtained with broad-spectrum recognition presented an affinity gradient consistent with toxicity levels (PHE > BUF > ABOB > MET). In addition, by simple dilution, the method allows monitoring of TDM and risk of biguanide poisoning in patients with type 2 diabetes. By extending the design strategy of biguanide antibodies to structurally complex macrolides, this study established a generalized hapten design approach that enables gradient-based recognition of complex analogue toxicity by antibodies through modulation of the hydrophobic exposure of key molecular sites. This provides new insights into the design of multi-residue ICA methods with customized requirements, which can be applied to other similar harmful drug analyses and provide a more universal tool for safeguarding public health risk monitoring.

## Author contributions

Jiarui Wang: investigation, writing-original draft. Lingling Guo: validation, funding acquisition. Chuanlai Xu: funding acquisition. Aihua Qu: writing-review & editing, validation. Hua Kuang: conceptualization, methodology, funding acquisition. Xinxin Xu: supervision, writing-review & editing.

## Conflicts of interest

No potential conflict of interest was reported by the authors.

## Supplementary Material

SC-OLF-D6SC03923E-s001

## Data Availability

The data supporting this article have been included as part of the supplementary information (SI). Supplementary information: detailed experimental procedures, characterization data, optimization results, and figures and tables. See DOI: https://doi.org/10.1039/d6sc03923e.
