# Supplementary material for: Guiding point-of-care therapeutic drug monitoring through structure–toxicity principles
Source: Chem Sci. 2026 Jul 2. Online ahead of print. doi: 10.1039/d6sc03923e (PMC13325025; doi:10.1039/d6sc03923e)
Supplement: SC-OLF-D6SC03923E-s001 [file SC-OLF-D6SC03923E-s001.pdf]

Supplementary Information (SI) for Chemical Science.
This journal is © The Royal Society of Chemistry 2026

Supporting information

Guiding Point-of-Care Therapeutic Drug Monitoring through Structure-Toxicity Principles

Table of Contents

Chemicals and materials

Computer-aided biguanides haptens design

Antibody modeling and molecular docking

Synthesis of biguanides haptens and attachment to proteins

Production and performance of mAbs

Sample preparation

ICA sensor assembly and detection principles

Characterization of the biguanides hapten and coupling to proteins

Synthesis of TIL Hapten and attachment to proteins

Figure S1 Structural characterization of H1. (A) LC-MS/MS characterization of H1, (B) 1 H NMR characterization of H1.

Figure S2. Identification of the molecular structure of H2/3/4. (A) Extraction chromatogram (EIC) and mass spectrometry of H2; (B) Extraction chromatogram (EIC) and mass spectrometry of H3; (C) Extraction chromatogram (EIC) and mass spectrometry of H4.

Figure S3. The Characterization of H1, H2, H3, H4, proteins and conjugates. (A) The Characterization of H1, BSA, OVA H1-OVA and H1-BSA; (B) The Characterization of H2, BSA, OVA H2-OVA and H2-BSA; (C) The Characterization of H3, BSA, OVA H3-OVA and H3-BSA; (D) The Characterization of H4, BSA, OVA H4-OVA and H4-BSA.

Figure S4. The antibody affinity constant of mAb 2G8.

Figure S5. Ramachandran plot.

Figure S6. Verify3D analysis of the mAb-2G8 protein model.

Figure S7. Optimization of the ICA strip for biguanides detection. (A) Optimization of GNPs-mAb dose; (B) Optimization of coating antigen concentration; and (C) Optimization of surfactants.

Figure S8. The results of the ICA strip curve for the determination of 4 biguanides in

PBS. (A) PHE, (B) BUF, (C) ABOB, (D) MET.

Figure S9. Results of the false-positive risk assessment in human serum samples from healthy individuals.

Figure S10. Synthesis and characterization of TIL Hapten and conjugates. (A) Synthesis pathways of Hapten, (B) LC-MS/MS characterization of Hapten, (C) The Characterization of Hapten, proteins and conjugates.

Figure S11. Optimization of the ICA strip for TIL and TYL detection. (A) Optimization of coating antigen concentration (0.08 mg/mL, 0.04 mg/mL) and GNPs-mAb dose (6 μg/mL, 3 μg/mL); (B) Optimization of surfactants (5% BSA, 5% PVP, 5% PEG, 5% R65). (N) 0 ng/mL of TIL, and (P) 200 ng/mL of TIL.

Table S1. Chromatographic conditions for analysis of biguanides by LC-MS/MS.

Table S2. Comparison of antibody affinity and LogP values for analytes.

Table S3. The specificity of the 2G8-mAb was determined by ic-ELISA.

Table S4. Standard curve equations and correlation coefficients for the determination of biguanides in human serum by ICA.

Reference

Chemicals and materials

Metformin, phenformin, buformin, moroxydine, tilmicosin, tylosin, chloriguanide, and cyclochloriguanide were purchased from Beijing Bailingwei Technology Co., Ltd. 4-Carboxybenzaldehyde, N-[2-(4-bromophenyl)ethyl]carbamic acid tert-butyl ester, potassium carbonate (K_2CO_3), glyoxylic acid, ethyl 6-bromohexanoate, and others were purchased from Sigma Aldrich (China). Bovine serum albumin (BSA), 1-(3-Dimethylaminopropyl)-3-ethylcarbodiimide hydrochloride (EDC), N-hydroxy succinimide (NHS), ovalbumin (OVA), N,N-dimethylformamide (DMF), and others were purchased from Beijing Bio-Reach Technology Co., Ltd. Freund's complete/incomplete adjuvant (FCA/FICA), horseradish peroxidase (HRP)-labeled goat anti-rat antibody, gelatin (derived from cold-water fish epidermis), and 3,3',5,5'-tetramethylbenzidine (TMB) were purchased from Jackson Immuno Sciences, Inc. (USA). All other chemical reagents were purchased from Sinopharm Group. All reagents were of analytical grade or higher purity.

The instruments used were UV-Vis spectrophotometer (Agilent Cary 60, Agilent Technologies, Inc., USA), fully digital NMR spectrometer (Avance III 400 MHz, Bruker AXS GmbH, Germany), and LC-MS/MS (Waters Quattro Premier XE, Waters Corporation, USA).

Computer-aided biguanides haptens design

To compare the structures and physicochemical properties of biguanide compounds with those of the hapten, the initial three-dimensional conformations of the molecules were manually constructed using GaussView 5.0 (Gaussian Wallingford, CT, USA) [1]. Subsequently, based on the optimized stable configuration, density functional theory calculations were performed using the B3LYP/6-31G(d) basis set to obtain precise electronic structural information. Unless otherwise specified, all calculations were performed in the gas phase. Vibrational frequency calculations were conducted at the same theoretical level to confirm that the optimized structure corresponds to a true local minimum on the potential energy surface, as indicated by the absence of imaginary frequencies. The conformation with the lowest electronic energy and no imaginary frequencies was selected for subsequent analysis. Building upon this, molecular overlay and pharmacophore analysis were further conducted using the Discovery Studio, 2024 (Accelrys Software, Inc., San Diego, CA, USA). Finally, the molecular surface electrostatic potential (ESP) energy and the occupied molecular orbital energy (E_{Homo} and E_{Lumo}) were analyzed and visualized with the Multiwfn (http://sobereva.com/multiwfn) and VMD software[2].

Antibody modeling and molecular docking

Following the methodology of Zou et al., the variable regions of the antibody heavy chain (VH) and light chain (VL) were successfully obtained through RNA extraction from hybridoma cells, cDNA synthesis, 5'RACE amplification, and plasmid construction/sequence validation[3]. The antibody sequences were aligned using Snapgene software to confirm the VL and VH regions. The VL and VH sequences were submitted to the AlphaFold2 server for antibody modelling prediction. The constructed antibody models were evaluated using Discovery Studio, 2024 and the MBI server (saves.mbi.ucla.edu/), yielding Ramachandran Plots and VERIFY-3D assessments to examine model validity. Within the Ramachandran plot, a higher number of amino acids within the yellow region indicates greater model reliability.

The highest-scoring model will be selected for subsequent molecular docking experiments. Variable regions were further processed using the abYsis server (http://www.abysis.org/abysis/sequence_input/key_annotation/key_annotation.cgi) to delineate CDR and framework domains. Molecular docking between the antibody and small-molecule antigen was performed with AutoDock-Vina software, with visualization of docking results achieved via PyMOL[4].

Synthesis of biguanides haptens and attachment to proteins

The synthetic pathways for H1, H2, H3, and H4 are detailed in the **Figure 1**. Haptens were dissolved in methanol for LC-MS/MS analysis. To further validate the successful preparation of the hapten, a 20 mg sample of the synthesized H1 was dissolved in an appropriate deuterated solvent and subjected to ^{1}H NMR for additional characterization.

H1: Briefly, a Heck coupling was performed by reacting *N*-Boc-4-bromophenethylamine (10 mmol) with methyl acrylate in DMF, using $Pd(PPh_3)_2Cl_2$ as a catalyst and K_2CO_3 as a base at 120 °C for 16 h. The resulting intermediate underwent catalytic hydrogenation over Pd/C in methanol, followed by Boc-deprotection using TFA in DCM. The obtained amine was then reacted with dicyandiamide in the presence of TMSOTf in 1,2-dichloroethane at 80 °C to introduce the biguanide group. After acidification with HCl, the final product was obtained through hydrolysis at 80 °C and purified via reversed-phase flash chromatography using an acetonitrile/water gradient.

H2: Synthesized from MET and p-formyl benzoic acid via the principle of aldehyde-amine condensation. Add 1.5 mL of distilled water and 30 μL of 1 mol/L hydrochloric acid to a beaker equipped with a magnetic stirring device. 0.2 mmol of p-formyl benzoic acid was added, and DMF was added dropwise until p-formyl benzoic acid was completely dissolved, and the pH was measured to be about 4.0. 0.2 mmol of MET was added under stirring, and the reaction was carried out at 60 °C overnight after dissolution with stirring. A white solid was obtained, the supernatant was discarded by centrifugation, washed three times with water, dried at 45 °C under a stream of nitrogen.

H3 was derivatized using MET and glyoxalin acid in the same way as H2, resulting in a yellow colloid.

H4: MET (0.1 mmol) was dissolved in anhydrous tetrahydrofuran (1.0 mL) under nitrogen atmosphere. K_2CO_3 (0.3 mmol) was then added and stirred for 10 min at room temperature. The resulting solution was reacted with ethyl 6-bromohexanoate (0.1 mmol) for 36 h at 70 °C, followed by hydrolysis with 1 M sodium hydroxide solution

for 6 h at 70 °C. The crude product was dried over anhydrous sodium sulfate and purified by rotary evaporation to give the final yellow product.

The immune response primarily depends on the immunogenicity and antigenicity of the antigen. Prior to conjugation to carrier proteins, small-molecule compounds require structural modification to prepare stable immunogens. All haptens bearing carboxyl groups were activated and conjugated via the carbodiimide method to BSA and OVA, serving as coating antigen and immunogen respectively[5]. Synthesis of immunogen and coating antigen: Haptens (0.005 mmol), EDC (0.015 mmol), and NHS (0.015 mmol) were dissolved in DMF (300 μL) and stirred overnight at room temperature. Upon reaction completion, the mixture was slowly added to carbonate-buffered saline (0.01 M, pH 9.5) containing BSA or OVA (6 mg), gently stirred overnight at room temperature, and finally dialyzed against PBS to yield the synthetic antigen. Finally, the complete antigen prepared was characterized using ultraviolet-visible spectrophotometry (UV-Vis).

Production and performance of mAbs

SPF-grade female BALB/c mice aged 6–8 weeks were selected. All animal experiments were approved by the Animal Welfare Committee of Jiangnan University and conducted in accordance with the committee's approved protocols and ethical guidelines, with the ethical approval number [JN. No2025 0515b1801125]. Mice were immunized using hapten-BSA as the immunizing antigen and hapten-OVA as the coating antigen[6]. A critical step in monoclonal antibody preparation is selecting positive mice that recognize target chemical residues. Following ic-ELISA analysis of serum from mice after the fifth booster immunization[7], we selected the most effectively immunized positive mice for subsequent cell fusion. After four subcloning rounds, a single cell line was obtained for mAb production.

Ascites preparation employs the in vivo induction method, specifically by inoculating hybridoma cells into the peritoneal cavity of mice to yield substantial quantities of antibody-containing ascites fluid. The specific operational steps are as follows: Seven days prior to the complete growth of the hybridoma cells, inject 0.5 mL of paraffin oil into the mouse's peritoneal cavity. On the day the tumor cells reach full growth, perform trypsin treatment to detach the cells. Transfer these to a 15 mL centrifuge tube and centrifuge at 1200 × g for 8 min. After discarding the supernatant, wash the cells twice with physiological saline, then centrifuge again under identical conditions. Discard the supernatant, resuspend the cell pellet in 2 mL physiological saline, and inject this suspension intraperitoneally into two separate mice. After seven days, ascites fluid was collected from mice exhibiting abdominal distension. This was centrifuged at 10,000 × g for 10 min, with the supernatant collected and stored at -20 °C.

The main subclasses of mouse antibodies are: IgG1, IgG2a, IgG2b, IgG3, IgM, and IgA. In order to identify the specific subtypes of these antibodies, commercially available mouse antibody subtype identification kits were used and specific procedures were followed[8]. The process of antibody sensitivity determination was as follows: firstly, working concentration screening was carried out: the antibody was diluted according to a gradient of 0.3, 0.1, 0.03, 0.01 μg/mL, and detected by ic-ELISA, and

the concentration with the OD_{450} value between 1.5-2.0 and the best inhibitory effect was selected as the working concentration[9]. Next, establish a standard curve: using this concentration for detection, prepare seven dilutions of the standard at 3-fold increments. The method used in this research was the PBS buffer, which served as the blank control, and the absorbance rates at OD_{450} nm were used to measure each sample. The most important sensitivity parameters, such as half-maximal inhibitory concentration (IC_{50}) and linear range of detection, were obtained by constructing a standard curve and carrying out a fitting analysis[10, 11].

The affinity to the binding of antibodies and antigens was measured with the following parameters: the concentration of coated antigens in solution was 0.3,0.1,0.03 μg/mL, the antibodies were diluted in gradient. Measurement of absorbance at OD_{450} nm was then done through ic-ELISA. The antibody affinity constant (Ka) is calculated using the following formula based on the molar concentration of antibody corresponding to the half-maximal signal value (ODmax/2) at different antigen concentrations: Ka = (n - 1) / 2 (n[Ab']t - [Ab]t), where n is the ratio of the neighboring antigen concentrations and [Ab]t is the molar concentration of antibody (mol/L) in ODmax/2. The final antibody affinity value represents the mean of the Ka values determined in multiple experimental measurements[12]. Cross-reactivity (CR) is an indication of the binding strength of the antibody with other than the target analyte analogues[13]. Its decision is based on the same steps as that of sensitivity assessment and is calculated as per the formula: CR (%) = Target IC_{50} / Biguanides IC_{50} × 100%.

ICA sensor assembly and detection principles

This study employed the trisodium citrate reduction method to synthesize gold nanoparticles (GNPs). Take 10 mL of GNPs solution, adjust the pH to 7.0 using 0.1 M K_2CO_3, add an appropriate amount of antibody, and gently shake for 45 min. Subsequently, add 1 mL of blocking solution and block at room temperature for 2 h. Centrifuge at 8000 × g for 15 min, discard the supernatant, and repeat the washing step twice. Finally, resuspend the pellet in 1 mL of resuspension buffer and store at 4 °C for later use.

The synthesis process of GNPs-mAb was as follows. The monoclonal antibody was diluted to 0.02 mg/mL using HEPES buffer (0.05 mol/L), and 16 μL of K_2CO_3 (0.2 mol/L) solution and diluted monoclonal antibody (5 μg or 10 μg) were added sequentially to 1 mL of colloidal gold solution, which was mixed homogeneously and then allowed to stand for 45 min, and then incubated for two hours by adding 50 μL of 10% BSA solution The reaction was incubated with 50 μL of 10% BSA solution for two hours to seal the unbound sites, and then centrifuged at 8500 × g for 45 min to remove the supernatant, and the precipitate was resuspended with 1.2 mL of colloidal gold resuspension solution. Finally, the solution was uniformly sprayed onto a binding pad and placed in a 37 °C oven to dry overnight.

The created ICA sensor system entails a sample pad, nitrocellulose (NC) membrane, absorbent pad, and PVC backing plate. Antigen (test line, T line) and goat anti-mouse antibody (control line, C line) are placed on the NC membrane[14]. The sample pad and absorbent pad are positioned at opposite ends, each overlapping the NC membrane by 1–2 mm. After assembly, the entire plate is cut into individual test strips 3–4 mm wide and stored in a dry environment.

The ICA sensor that we have made can work based on the concept of the classical competitive assay[15, 16]. When the sample is applied, the liquid moves forward through the test strip by capillary action. In the case of negative samples, the GNPs-mAb bind to the antigen immobilized on the T line during migration, and produce a specific red band. The free target analytes of the positive samples in competition with limited GNPs-mAb to bind to T-line antigen occur in positive samples. With a rise in target concentration, additional GNPs-mAbs are effectively blocked from binding to the T line, which results in a gradual decrease in the color of the T line until it becomes completely blocked at high concentration. The C line is a quality control in which a secondary antibody that is able to bind to the GNPs-mAb is applied on the line. It must show a red color irrespective of the outcome of the test, which will show that the strip is functioning properly. A quantitative calibration curve was constructed by plotting the strip T/C signal against analyte concentration.

Sample preparation

Human serum samples were obtained from the Affiliated Hospital of Jiangnan University. This study was conducted in accordance with the Declaration of Helsinki and approved by the Ethics Committee of Affiliated Hospital of Jiangnan University (Approval No. LS2024356). Informed consent was obtained from all individual participants included in the study. All samples were anonymized and coded to ensure the protection of personal privacy and data confidentiality. Human serum samples were diluted with PBS (containing 1% ON870) for the assay.

Instrumental detection employed LC-MS/MS[20], with the method slightly modified from prior studies (**Table S1**).

Characterization of the biguanides hapten and coupling to proteins

H1-H4 were identified by LC-MS/MS. Chromatograms in **Figure S1 and Figure S2** show retention times of 0.81, 0.86, 1.84, and 2.89 min, respectively. Corresponding molecular ion peaks in the mass spectra appeared at m/z 278, 262, 244, and 186 $[M+1]^+$, consistent with the molecular masses of the hapten. This confirmed the successful derivatisation of the hapten. The ^1H NMR spectrum of H1 is shown in **Figure S1**, with the following analysis: (400 MHz, D_2O): δ 7.16 (s, 4H), 3.42 (t, J = 6.8 Hz, 2H), 2.84–2.77 (m, 4H), 2.58 (t, J = 7.6 Hz, 2H).

The synthetic antigen was validated via ultraviolet-visible spectroscopy (UV-VIS), with results presented in **Figure S3**. Comparing the absorption characteristics of haptens before and after conjugation with carrier proteins (BSA, OVA) across the 200–800 nm wavelength range revealed varying degrees of shift in the artificial antigen's absorption peaks relative to the carrier proteins' maximum absorption peaks (278 nm, 280 nm). This shift indicates successful conjugation of the hapten to the carrier protein.

Synthesis of TIL Hapten and attachment to proteins

TIM (0.1 mmol) was weighed and dissolved with O-(carboxymethyl)hydroxylamine (CMO, 0.3 mmol) in anhydrous pyridine and reacted with stirring in a water bath at 70 °C for 12 h. The carbonyl group was allowed to fully

react with CMO to form the oxime derivative. The reaction solution was cooled and the pyridine was removed by evaporation under reduced pressure. The residue was dissolved in distilled water and the pH was adjusted to 4.0-5.0 with 0.1 M HCl to precipitate. The precipitate was extracted with ethyl acetate, and the organic phase was dried over anhydrous sodium sulfate and rotary evaporated to obtain the crude product, which was finally purified by silica gel column. The final product was purified by silica gel column. The successful synthesis of hapten was confirmed by LC-MS. The synthesis of the artificial antigen was verified by UV-Vis as in the case of biguanide antigen.

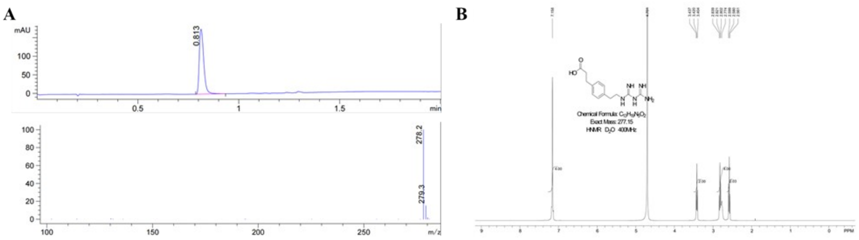

Figure S1. Structural characterization of H1. (A) LC-MS/MS characterization of H1, (B) 1 H NMR characterization of H1.

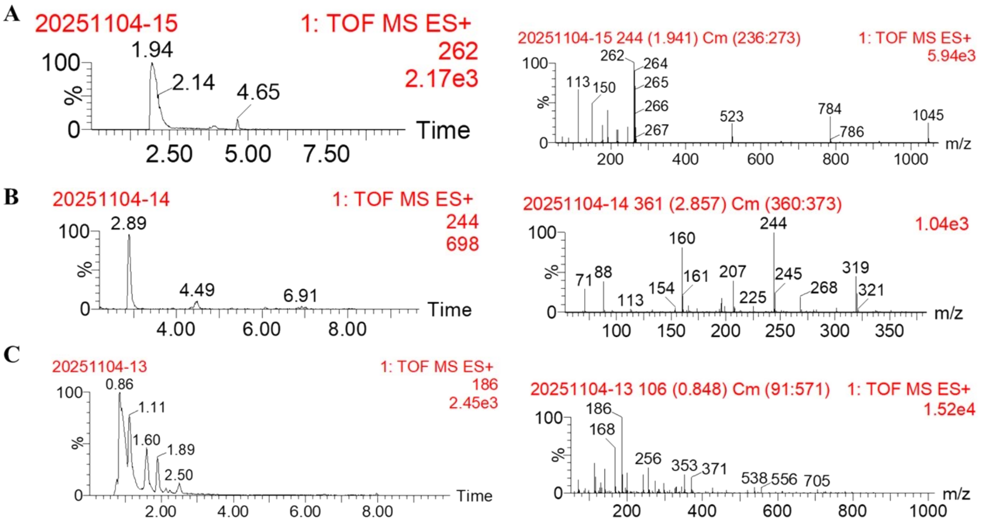

Figure S2. Identification of the molecular structure of H2/3/4. (A) Extraction chromatogram (EIC) and mass spectrometry of H2; (B) Extraction chromatogram (EIC) and mass spectrometry of H3; (C) Extraction chromatogram (EIC) and mass spectrometry of H4.

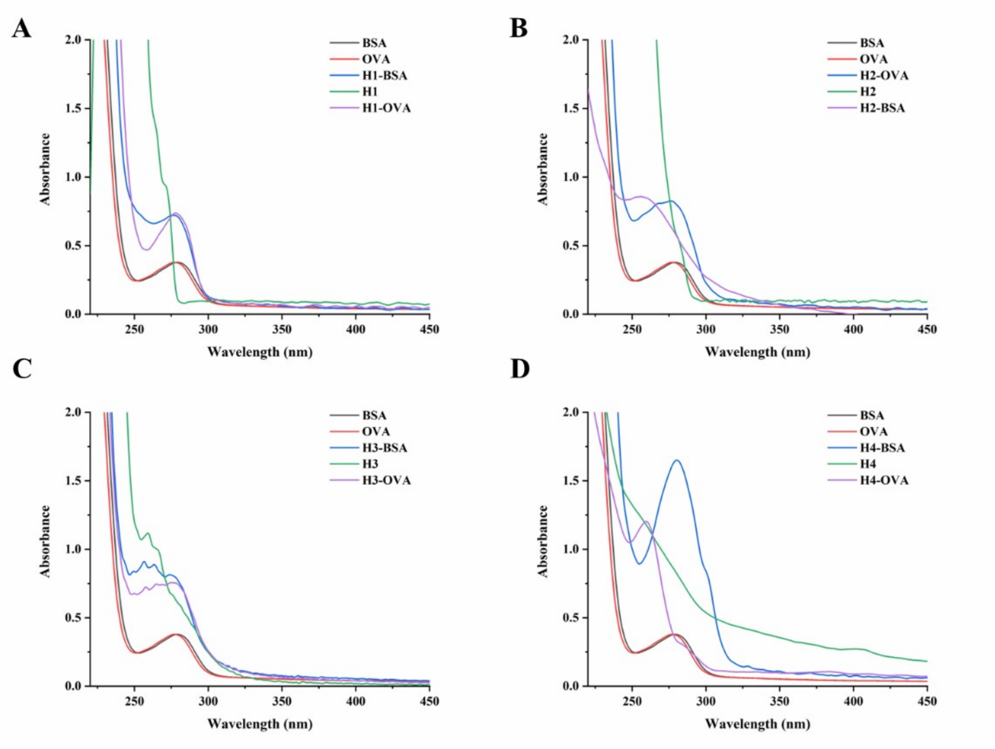

Figure S3. The Characterization of H1, H2, H3, H4, proteins and conjugates. (A) The Characterization of H1, BSA, OVA H1-OVA and H1-BSA; (B) The Characterization of H2, BSA, OVA H2-OVA and H2-BSA; (C) The Characterization of H3, BSA, OVA H3-OVA and H3-BSA; (D) The Characterization of H4, BSA, OVA H4-OVA and H4-BSA.

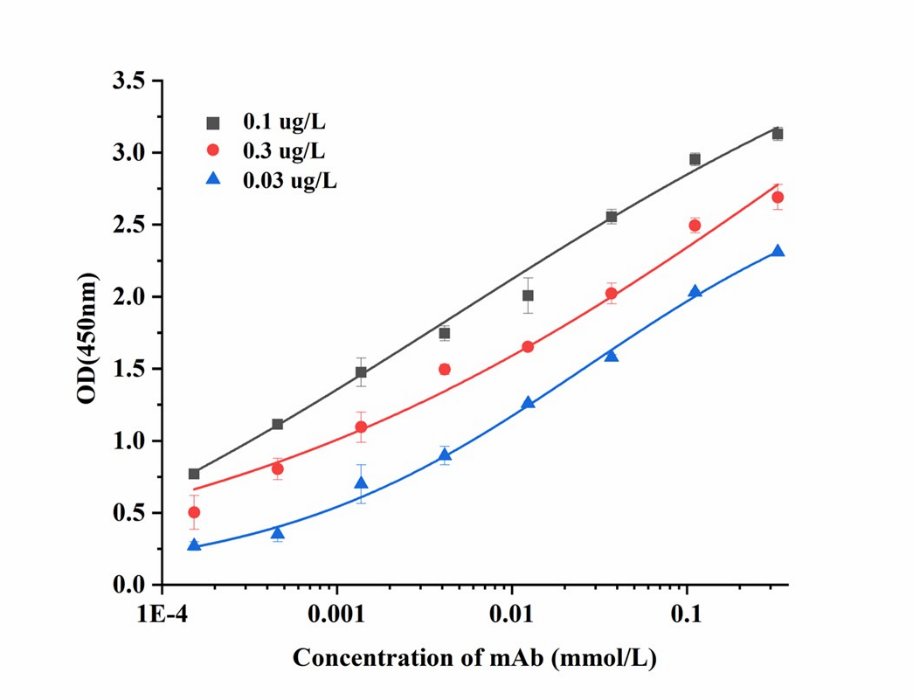

Figure S4. The antibody affinity constant of mAb 2G8.

Comments: The antibody affinity constant (Ka) is calculated using the following formula based on the molar concentration of antibody corresponding to the half-maximal signal value (ODmax/2) at different antigen concentrations: Ka = (n - 1) / 2 (n[Ab’]t - [Ab]t), where n is the ratio of the neighboring antigen concentrations and [Ab]t is the molar concentration of antibody (mol/L) in ODmax/2. The final antibody affinity value represents the mean of the Ka values determined in multiple experimental measurements. Three concentration gradients were set for the antigen in order to accurately calculate the antibody affinity and verify the validity of the binding model by fitting a binding curve.

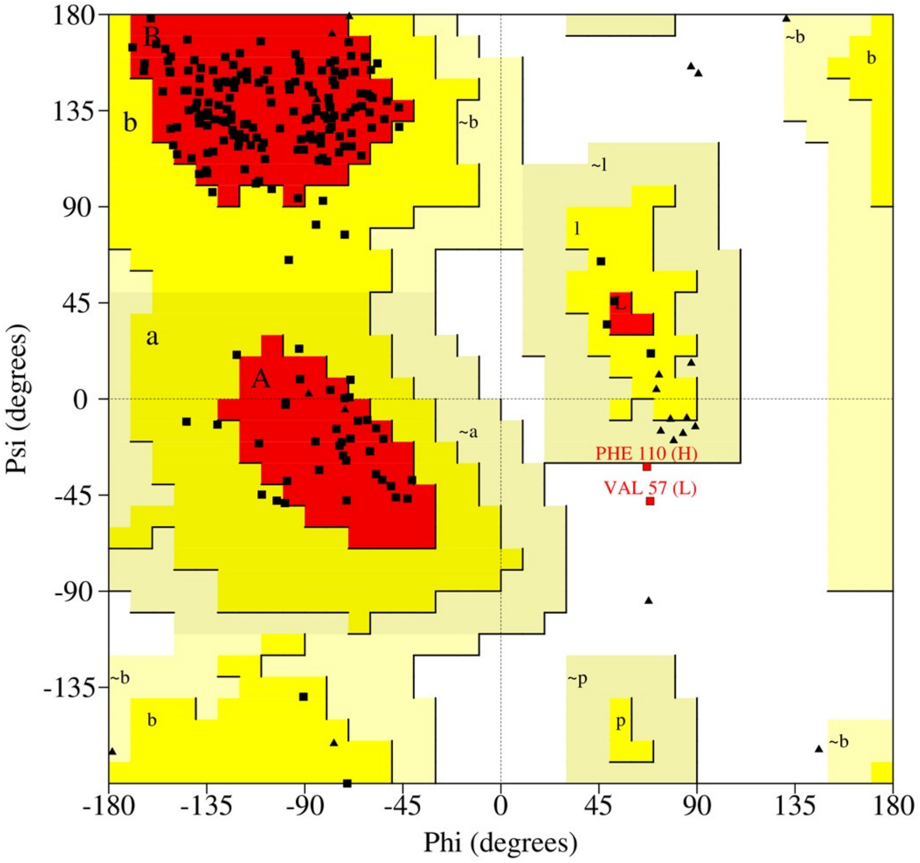

Figure S5. Ramachandran plot.

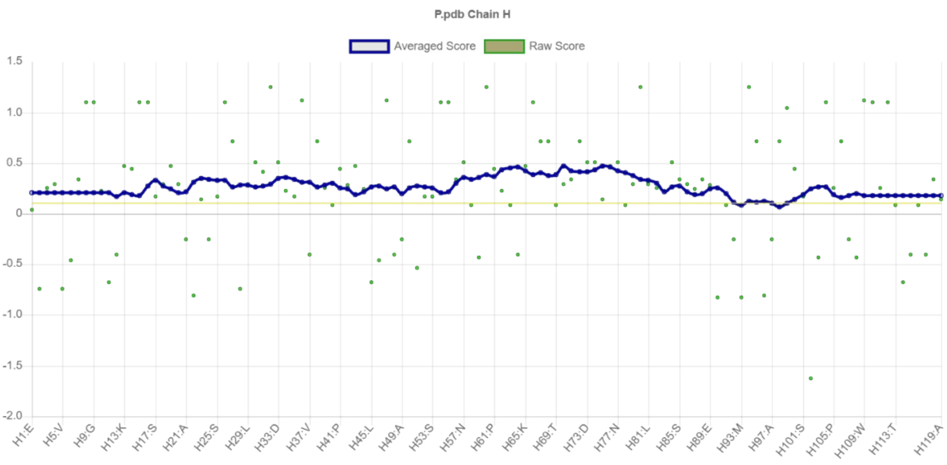

Figure S6. Verify3D analysis of the mAb-2G8 protein model.

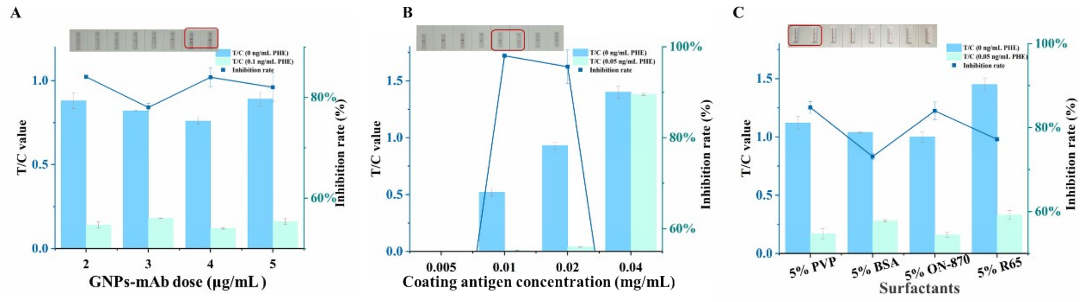

Figure S7. Optimization of the ICA strip for biguanides detection. (A) Optimization of GNPs-mAb dose; (B) Optimization of coating antigen concentration; and (C) Optimization of surfactants.

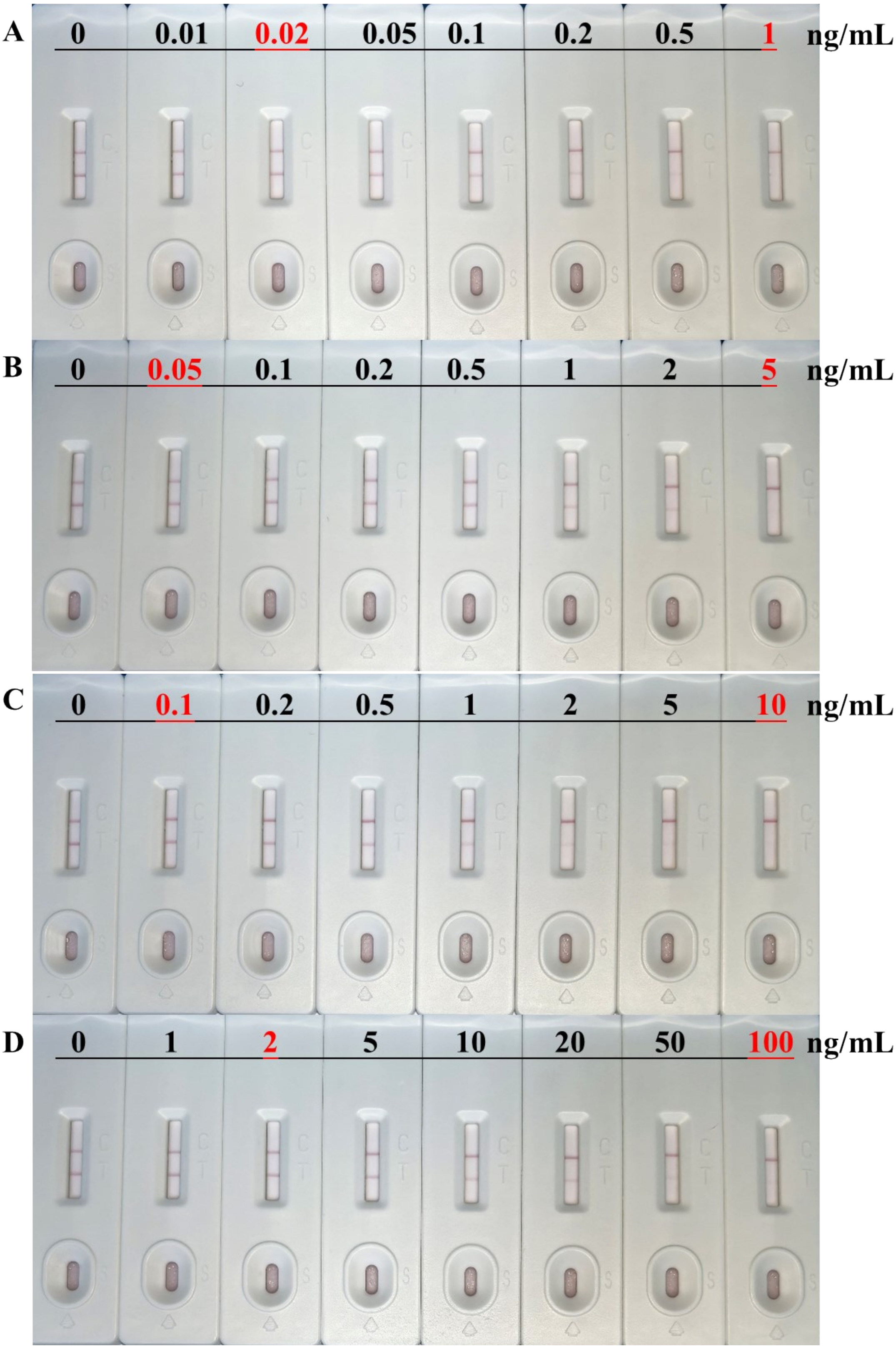

Figure S8. The results of the ICA strip curve for the determination of 4 biguanides in PBS. (A) PHE, (B) BUF, (C) ABOB, (D) MET.

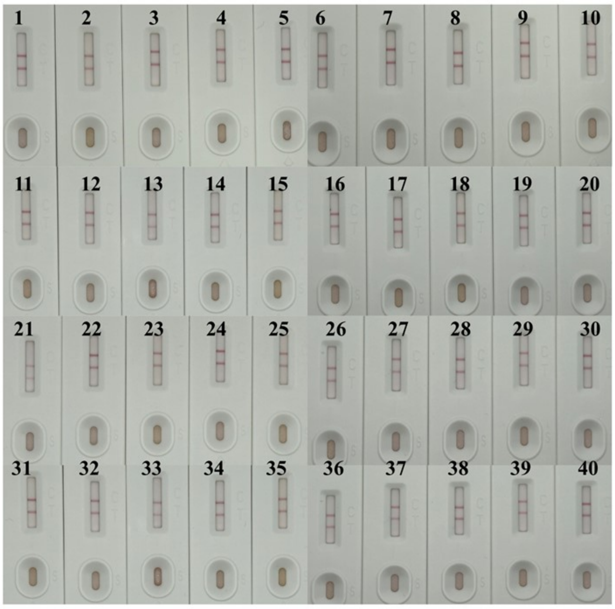

Figure S9. Results of the false-positive risk assessment in human serum samples from healthy individuals.

A B C

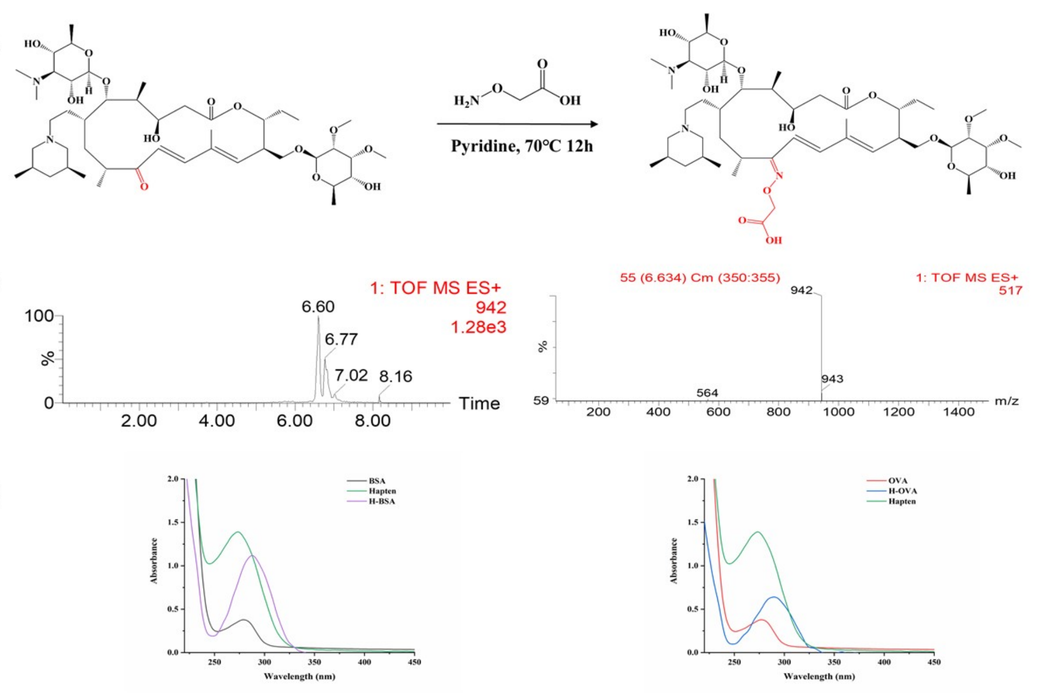

Figure S10. Synthesis and characterization of TIL Hapten and conjugates. (A) Synthesis pathways of Hapten, (B) LC-MS/MS characterization of Hapten, (C) The Characterization of Hapten, proteins and conjugates.

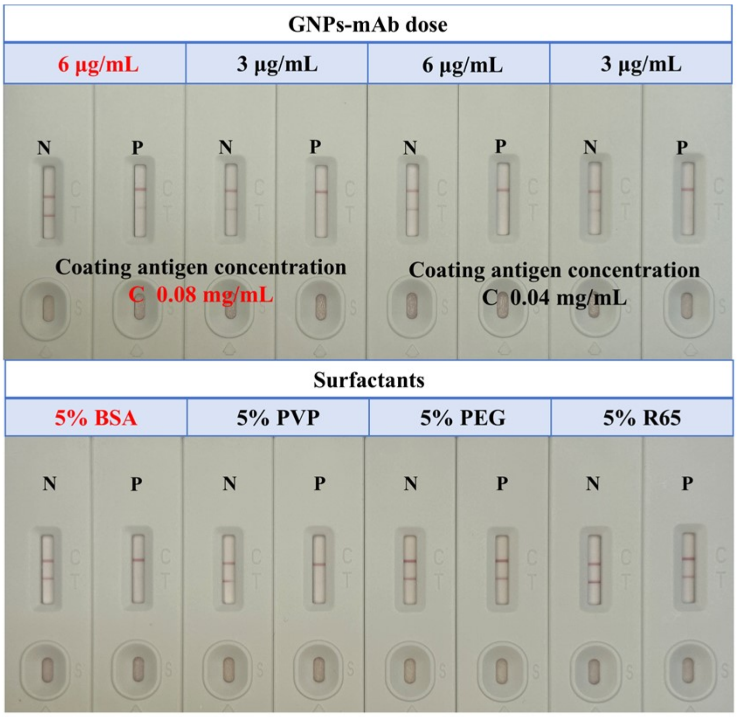

Figure S11. Optimization of the ICA strip for TIL and TYL detection. (A) Optimization of coating antigen concentration (0.08 mg/mL, 0.04 mg/mL) and GNPs-mAb dose (6 μg/mL, 3 μg/mL); (B) Optimization of surfactants (5% BSA, 5% PVP, 5% PEG, 5% R65). (N) 0 ng/mL of TIL, and (P) 200 ng/mL of TIL.

Table S1. Chromatographic conditions for analysis of Biguanides by LC-MS/MS.

	Chromatographic conditions		
Chromatographic Column	Agilent Poroshell 120 SB-Aq (2.1×100 mm, 2.7 μm)		
	Column temperature: 30 °C		
Mobile Phase	A: 0.1% FA in water		
	B: Methanol		
Gradient Profile	Time (min)	Percentage A (%)	Percentage B (%)
	0.00	100.00%	0.00%
	2.00	100.00%	0.00%
	2.50	60.00%	40.00%
	4.00	25.00%	75.00%
	5.00	25.00%	75.00%
	7.00	100.00%	0.00%
Injection Volume	10 μL		

Table S2. Comparison of antibody affinity and LogP values for analytes.

Analytes	IC_{50} (ng/mL)	LogP
Phenformin	0.11	1.78
Buformin	0.25	1.01
Metformin	3.35	0.15
Moroxydine	0.54	-0.25

Table S3. The specificity of the 2G8-mAb was determined by ic-ELISA.

Analogs	IC_{50} (ng/mL)	CR (%)
Phenformin	0.11	491
Buformin	0.25	216
Moroxydine	0.54	100
Metformin	3.35	16.1
Chloroguanide	>500	<1
Chlorhexidine	>500	<1
Cyclochloroguanide	>500	<1
Polyhexamethylene biguanide	>500	<1

Table S4. Standard curve equations and correlation coefficients for the determination of biguanides in human serum by ICA.

Biguanides	Correlation coefficients (R^2)	Standard curve equations
Phenformin	0.997	y=-0.04+0.99/(1+(x/0.163)^1.00)
Buformin	0.993	y=-0.06+1.05/(1+(x/0.97)^1.16)
Moroxydine	0.991	y=-0.56+1.55/(1+(x/23.52)^0.68)
Metformin	0.994	y=0.01+1.08/(1+(x/45.87)^1.12)

Reference

1. Zhang, J.; Lu, T. Efficient evaluation of electrostatic potential with computerized optimized code. *Physical Chemistry Chemical Physics* **2021**, *23* (36), 20323–20328. DOI: 10.1039/d1cp02805g.

2. Lu, T.; Chen, F. W. Multiwfn: A multifunctional wavefunction analyzer. *Journal of Computational Chemistry* **2012**, *33* (5), 580–592. DOI: 10.1002/jcc.22885.

3. Zou, R. B.; Guo, Y. H.; Wang, Y.; Lu, X. Y.; Ma, Z. J.; Shou, L. F.; Liu, Y. H.; Zhu, G. N.; Guo, Y. R. Insights into the Binding Profile of Anti-chlorpyrifos Recombinant Antibodies: From Computational Simulation to Immunoassay Validation. *Analytical Chemistry* **2023**, *95* (30), 11287–11295. DOI: 10.1021/acs.analchem.3c01355.

4. Eberhardt, J.; Santos-Martins, D.; Tillack, A.; Forli, S. AutoDock Vina 1.2.0: New Docking Methods, Expanded Force Field, and Python Bindings. *Journal of Chemical Information and Modeling* **2021**, *61* (8), 3891–3898. DOI: 10.1021/acs.jcim.1c00203.

5. Gao, J. X.; Zhang, T. Y.; Fang, Y. H.; Zhao, Y.; Yang, M.; Zhao, L.; Li, Y.; Huang, J.; Zhu, G. N.; Guo, Y. R. On-site rapid detection of multiple pesticide residues in tea leaves by lateral flow immunoassay. *Journal of Pharmaceutical Analysis* **2024**, *14* (2), 276–283. DOI: 10.1016/j.jpha.2023.09.011.

6. Sekizuka, R.; Kitsunezuka, K.; Miyano, N.; Inoue, H.; Miyasaka, A.; Nakagawa, H.; Oonaka, K.; Kushiro, M.; Miyake, S. Rapid and easy determination of mycotoxin nivalenol in wheat and barley by direct competitive enzyme-linked immunosorbent assay. *Food Control* **2025**, *177*, 111432. DOI: 10.1016/j.foodcont.2025.111432.

7. Peng, P.; Liu, C.; Li, Z.; Xue, Z.; Mao, P.; Hu, J.; Xu, F.; Yao, C.; You, M. Emerging ELISA derived technologies for in vitro diagnostics. *Trac-Trends in Analytical Chemistry* **2022**, *152*, 116605. DOI: 10.1016/j.trac.2022.116605.

8. Kassir, A. A.; Cheignon, C.; Charbonnière, L. J. Exploitation of Luminescent Lanthanide Nanoparticles for a Sensitivity-Enhanced ELISA Detection Method. *Analytical Chemistry* **2024**, *96* (5), 2107–2116. DOI: 10.1021/acs.analchem.3c04821.

9. Cho, C.; Kim, J.; Padalkar, N.; Reddy, Y.; Park, T.; Park, J.; Park, J. Nanozyme-assisted molecularly imprinted polymer-based indirect competitive ELISA for the detection of marine biotoxin. *Biosensors & Bioelectronics* **2024**, *255*. DOI: 10.1016/j.bios.2024.116269.

10. Zou, R. B.; Guo, Y. H.; Chen, Y.; Zhao, Y.; Zhao, L.; Zhu, G. N.; Liu, Y. H.; Peters, J.; Guo,

Y. R. Computer-aided profiling of a unique broad-specific antibody and its application to an ultrasensitive fluoroimmunoassay for five N-methyl carbamate pesticides. *Journal of Hazardous Materials* **2022**, *426*. DOI: 10.1016/j.jhazmat.2021.127845.

11. Urusov, A.; Gubaidullina, M.; Petrakova, A.; Zherdev, A.; Dzantiev, B. A new kind of highly sensitive competitive lateral flow immunoassay displaying direct analyte-signal dependence. Application to the determination of the mycotoxin deoxynivalenol. *Microchimica Acta* **2018**, *185* (1), 29. DOI: 10.1007/s00604-017-2576-6.

12. Li, X.; Wu, A.; Sun, M.; Song, S.; Kuang, H.; Xu, C.; Wu, X. Simulation-assisted hapten screening for sensitive on-site and visual detection of diazinon pesticide in environmental water and food samples. *Chemical Engineering Journal* **2025**, *505*, 159017. DOI: 10.1016/j.cej.2024.159017.

13. Wang, Y.; Zhao, Y.; Li, J.; Zhao, X.; Liu, J.; Wang, S.; Wu, H.; Shi, G. On-site monitoring of atrazine in edible oils: Integrating reverse phase transfer extraction with lateral flow immunoassay for rapid screening. *Analytica Chimica Acta* **2025**, *1375*, 344560. DOI: 10.1016/j.aca.2025.344560.

14. Ghoshdastidar, S.; Gangula, A.; Kainth, J.; Saranathan, S.; Elangovan, A.; Afrasiabi, Z.; Hainsworth, D. P.; Upendran, A.; Kannan, R. Plate-Adherent Nanosubstrate for Improved ELISA of Small Molecules: A Proof of Concept Study. *Analytical Chemistry* **2020**, *92* (16), 10952–10956. DOI: 10.1021/acs.analchem.0c01441.

15. Wang, Z.; Zheng, S.; Wang, C.; Zhang, L.; Liu, Y.; Wu, X.; Wang, S. A novel competitive color-tone change fluorescence immunochromatographic assay for the ultrasensitive detection of pesticide and veterinary drug residues. *Sensors and Actuators B-Chemical* **2024**, *417*, 136125. DOI: 10.1016/j.snb.2024.136125.

16. Pedreira-Rincón, J.; Rivas, L.; Comenge, J.; Skouridou, V.; Camprubi-Ferrer, D.; Muñoz, J.; O'Sullivan, C.; Chamorro-Garcia, A.; Parolo, C. A comprehensive review of competitive lateral flow assays over the past decade. *Lab on a Chip* **2025**, *25* (11), 2578–2608. DOI: 10.1039/d4lc01075b.

17. Zhang, Y. Y.; Zhang, X. X.; Chen, C. T.; Wang, J. X.; Wei, F. C.; Liu, H. Q.; Du, F. K.; Jiang, L. R.; Zhu, W. W.; Tan, X. C.; et al. β-Lactoglobulin Amyloid Fibrils-Poly(vinyl alcohol)-AgNPs Hydrogel as a Surface-Enhanced Raman Substrate for All-in-One Rapid Separation, Enrichment, and Detection of Small Molecules in Food Samples. *Analytical Chemistry* **2025**, *97* (30), 16491–16505. DOI: 10.1021/acs.analchem.5c02529.

18. Rodríguez, E.; Li, Y. Y.; Vaniya, A.; Shih, P. M.; Fiehn, O. Alternative Identification of Glycosides Using MS/MS Matching with an In Silico-Modified Aglycone Mass Spectra Library. *Analytical Chemistry* **2023**, *95* (28), 10618–10624. DOI: 10.1021/acs.analchem.3c00957.

19. Chen, P. X.; Liu, Z. W.; Pan, K. L.; Lei, Y.; Yu, X. Q.; Huang, X. N.; Chen, J. H.; Lei, H. T. Rational Hapten Design for the Immunochromatographic Assay of Yohimbine, an Emerging Adulterant in Food. *Journal of Agricultural and Food Chemistry* **2025**, *73* (43), 27669–27679. DOI: 10.1021/acs.jafc.5c05567.

20. Wang, Z. D.; Wang, X. R.; Wang, Y. H.; Wu, C. L.; Zhou, J. H. Simultaneous determination of five antiviral drug residues and stability studies in honey using a two-step fraction capture coupled to liquid chromatography tandem mass spectrometry. *Journal of Chromatography A* **2021**, *1638*, 461890. DOI: 10.1016/j.chroma.2021.461890.
